# Tolerability and effectiveness of palbociclib in older women with metastatic breast cancer

**DOI:** 10.1007/s10549-024-07312-y

**Published:** 2024-04-16

**Authors:** Joosje C. Baltussen, Simon P. Mooijaart, Annelie J.E. Vulink, Danny Houtsma, Wendy M. Van der Deure, Elsbeth M. Westerman, Hendrika M. Oosterkamp, Leontine E.A.M.M. Spierings, Frederiek van den Bos, Nienke A. de Glas, Johanneke E.A. Portielje

**Affiliations:** 1 Department of Medical Oncology, Leiden University Medical Center, Albinusdreef 2, Leiden, Postzone C7-Q, P.O. Box 9600 RC, Leiden, the Netherlands; 2 Department of Gerontology and Geriatrics, Leiden University Medical Center, Leiden, the Netherlands; 3 LUMC Center for Medicine for Older People, LUMC, Leiden, The Netherlands; 4 Department of Medical Oncology, Reinier de Graaf Hospital, Delft, the Netherlands; 5 Department of Internal Medicine, Haga Hospital, The Hague, the Netherlands; 6 Department of Internal Medicine, Groene Hart Hospital, Gouda, the Netherlands; 7 Department of Clinical Pharmacy, Haaglanden Medical Center, The Haque, The Netherlands; 8 Department of Medical Oncology, Haaglanden Medical Center, The Haque, The Netherlands; 9 Department of Internal Medicine, Alrijne Hospital, Leiderdorp, The Netherlands

**Keywords:** Palbociclib, Older patients, Metastatic breast cancer, Tolerability, Toxicity, Real-world data

## Abstract

**Purpose:**

Palbociclib has become the standard of care for estrogen receptor-positive (ER+), human epidermal growth factor receptor 2 negative (HER2-) metastatic breast cancer, but real-world evidence in older women remains scarce. Therefore, we investigated tolerability of palbociclib in older women with metastatic breast cancer.

**Methods:**

Consecutive women aged ≥ 70 with ER+/HER2- metastatic breast cancer, treated with palbociclib in any treatment line in six hospitals, were included. Primary endpoint was grade ≥ 3 palbociclib-related toxicity. Predictors of toxicity were identified using logistic regression models. Progression-free survival (PFS) and overall survival (OS) were estimated using Kaplan Meier.

**Results:**

We included 144 women with a median age of 74 years. Grade 3–4 toxicity occurred in 54% of patients, of which neutropenia (37%) was most common. No neutropenic fever or grade 5 toxicity occurred. Dose reduction during treatment occurred in 50% of patients, 8% discontinued treatment due to toxicity and 3% were hospitalized due to toxicity. Polypharmacy (odds ratio (OR) 2.50; 95% confidence interval (CI) 1.12–5.58) and pretreatment low leukocytes (OR 4.81; 95% CI 1.27–18.21) were associated with grade 3–4 toxicity, while comorbidities were not. In first-line systemic therapy, median PFS was 12 months and median OS 32 months. In second-line, median PFS was 12 months and median OS 31 months.

**Conclusion:**

Although grade 3–4 toxicity and dose reductions occurred frequently, most were expected and managed by dose reductions, showing that palbociclib is generally well tolerated and thus represents a valuable treatment option in the older population.

**Supplementary Information:**

The online version contains supplementary material available at 10.1007/s10549-024-07312-y.

## Introduction

Breast cancer is a common disease among older women, with over 30% of new cases diagnosed in patients aged ≥ 70 years [1]. Yet, they are still underrepresented in pivotal trials investigating novel therapies [2]. Furthermore, previous studies demonstrated that older patients included in breast cancer trials do not represent the general older population, as they have less comorbidities, a better socioeconomic status and less aggressive disease [3, 4]. This results in limited data about the tolerability and benefit of anticancer treatment for most older women seen in daily practice.

Of all older women diagnosed with breast cancer, 80% have estrogen receptor-positive (ER+)/ human epidermal growth factor receptor 2-negative (HER2-) breast cancer [5]. In the metastatic setting, sequential endocrine therapy has been the standard treatment for this tumor type for decades, but many patients will develop acquired resistance to endocrine therapy at some point and are then candidates for chemotherapy. The introduction of cyclin-dependent kinases 4 and 6 (CDK4/6) inhibitors such as palbociclib has transformed the treatment landscape of metastatic ER+/HER2- breast cancer [6]. Clinical trials have demonstrated a benefit in progression-free survival (PFS) of 7–10 months [7–9] and, in some trials, a prolongation in overall survival (OS) [10] for the combination of palbociclib and endocrine therapy compared to endocrine therapy alone. This benefit led to rapid approval and recommendation of its use as first- or second-line treatment option by international guidelines [11].

With palbociclib being commonly used in older women living with frailty and multimorbidity, real-world data are needed to understand its safety in clinical practice [12]. Yet, data derived from real-life settings remain scarce. Therefore, this study assessed the treatment tolerability of palbociclib in older women with ER+/HER- metastatic breast cancer using real world data.

## Methods

This retrospective, multicenter cohort study was conducted in six Dutch hospitals and received approval from all institutional review boards of the participating hospitals. No formal dedicated informed consent was required, but all patients had approved use of their data by the opt-out procedure.

We included all consecutive women aged ≥ 70 years with ER+/HER2- metastatic breast cancer, treated with palbociclib between January 2016 and July 2022. Palbociclib could be administered in combination with anti-estrogen therapy or aromatase inhibitors, during any line of endocrine therapy. Only patients with a minimal follow-up time of 6 months or death before that date were included.

Data were collected from digital patient files. Patient characteristics included comorbidity, polypharmacy (using ≥ 5 medications), WHO performance status, Body Mass Index (BMI), living situation and baseline leukocytes. Comorbidity was measured using the Charlson Comorbidity Index (CCI) [13]. Leukocytes were categorized with a cut-off < 5 10^9^/L [14]. Tumor- and treatment characteristics included the number and location of metastatic sites, upfront dose reduction, type and line of endocrine therapy and prior chemotherapy use.

Primary endpoint was grade ≥ 3 palbociclib-related toxicity, defined by the Common Terminology Criteria for Adverse Event v5.0 [15]. Secondary endpoints included dose reduction or dose delay during treatment, treatment discontinuation and unplanned hospital admissions. PFS (time from start palbociclib to date of radiologic or biochemical progression or date of death as a result of any cause, whichever occurred first) and OS (time from start of palbociclib to date of death) were also calculated.

### Statistical analyses

Descriptive statistics were calculated using median and interquartile ranges for continuous data and frequencies and percentages for categorical data. Median follow-up time was calculated using the reverse Kaplan-Meier method [16]. To identify predictors of grade ≥ 3 toxicity, uni- and multivariable regression models were calculated using odds ratios (OR) and their 95% confidence intervals (CI). Clinically relevant predictors (bone only disease, line of therapy, upfront dose reduction, WHO status and comorbidities) and those with a *p* < 0.1 were added to the multivariable model.

Median PFS and OS with their 95% CI were estimated using the Kaplan-Meier method. To take into account time-related bias when studying patients treated in different treatment lines, survival analyses were stratified by line of systemic therapy. To investigate whether the survival of women who received upfront dose reduction or a dose reduction within the first three months was worse compared to those treated with full-dose, we performed a sensitivity analysis in which we stratified survival by dose reduction during or before treatment initiation versus no dose reduction. To reduce the bias that patients with a longer PFS time have had a higher probability of receiving a dose reduction, we only considered dose reduction within the first three months after treatment initiation.

Analyses were performed in SPSS v29 and figures were created using GraphPad Prism 9.3.1. P-values were 2-sided and a p-value < 0.05 was considered statistically significant.

## Results

We included 144 older women treated with palbociclib between January 2016 and July 2022. The median follow-up time from palbociclib initiation was 32 months (IQR 18–47). The median age of the participants was 74 years (IQR 72–78) (Table 1). Approximately 73% had recurrent or progressive disease and 19% had bone metastases only. Polypharmacy was seen in 57% and a CCI of ≥ 1 in 39%. Of all women, 47 (33%) received palbociclib in first-line, 53 (37%) in second-line and 44 (31%) in third-, fourth- or fifth-line. Upfront dose reduction was performed in 10 (7%) patients. Median treatment duration of palbociclib was 9 months (IQR 5–19) (first-line; 10 months (IQR 5–18), second-line; 9 months (IQR 4–18), third-, fourth- or fifth-line; 10 months (IQR 4–20)).

**Table 1:** Baseline characteristics (*N* = 144)

	Variable	*N (%)*
Age (years)	Median (IQR)	74 (72–78)
	70–74	75 (52.1)
	75–79	51 (35.4)
	≥ 80	18 (12.5)
Presentation	Newly diagnosed disease	39 (27.1)
	Recurrent or progressive disease	105 (72.9)
Metastases	Bone involvement	114 (79.2)
	Lung involvement Liver involvement	55 (38.2) 50 (34.7)
	Bone only	27 (18.8)
Endocrine therapy	Aromatase inhibitor	42 (29.2)
	Anti-estrogen	102 (70.8)
Line of endocrine therapy	1	47 (32.6)
	2	53 (36.8)
	≥ 3	44 (30.6)
Starting dose palbociclib	125 mg (standard dose)	134 (93.1)
	100 mg 75 mg	9 (6.3) 1 (0.7)
Prior chemotherapy	Yes	16 (11.1)
Concurrent radiotherapy	Yes	20 (13.9)
Baseline leukocytes	Mean (SD)	7.58 (3.15)
	Normal (> 5 10^9^/L)	113 (78.5)
	Low (≤ 5 10^9^/L)	20 (13.9)
	Unknown	11 (7.6)
WHO performance status	0 1 2 3 Not recorded	27 (18.8) 39 (27.1) 19 (13.2) 2 (1.4) 57 (39.6)
BMI	Mean (SD)	26.7 (4.9)
	< 20 kg/m²	7 (4.9)
	20–24.9 kg/m²	41 (28.5)
	25–30 kg/m²	44 (30.6)
	> 30 kg/m²	21 (14.6)
	Not recorded	31 (21.5)
Charlson comorbidity index	0	89 (61.4)
	1	28 (19.3)
	2	19 (13.1)
	3	8 (5.5)
N of medications	0–4	62 (43.1)
	≥ 5	82 (56.9)
Living situation	With others	66 (45.8)
	Alone	55 (38.2)
	Institutionalized*	3 (2.1)
	Unknown	20 (13.9)

*Abbreviations* IQR; interquartile range, N: number, SD; standard deviation. *Living in a nursing home or a rehabilitation center

### Tolerability

**Fig. 1:**
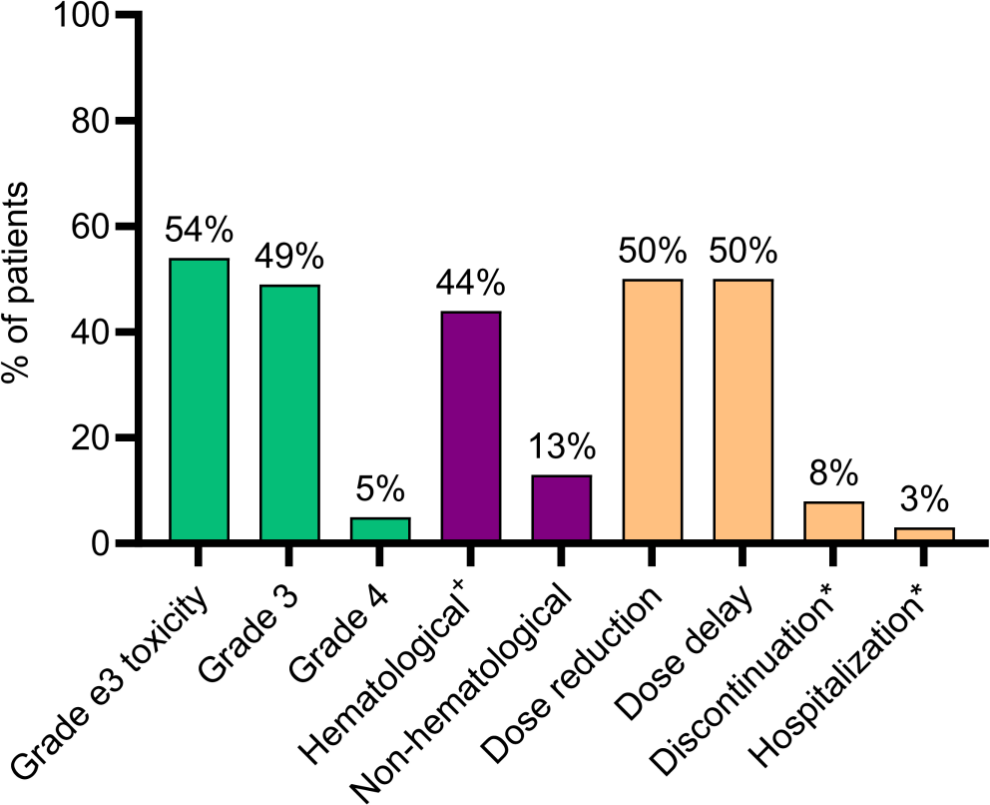
Treatment outcomes after palbociclib of all 144 patients. No patients in our cohort had grade 5 toxicity. ^+^Of the 78 patients with grade 3–5 hematological toxicity, 73 (94%) only had grade 3–4 neutropenia or leukopenia and 5 (6%) had neutropenia combined with thrombocytopenia or anemia. *Early treatment discontinuation and unplanned hospitalizations due to palbociclib-related toxicity

Of all women, 78 (54%) developed grade 3–4 palbociclib-related toxicity, of which 63 (44%) had hematological and 18 (13%) non-hematological toxicities (Fig. 1). Neutropenia (55 women, 38%) and leukopenia (10 women, 7%) were the most common hematological toxicities and fatigue (9 women, 6%) the most common non-hematological toxicity (Table S1, Online Resource). Grade ≥ 3 febrile neutropenia or treatment-related death was not reported. Of the 78 women who experienced a grade 3–4 toxicity, the toxicity led to a dose reduction in 28%, to dose delay in 18%, to reduction and dose delay in 26%, and to treatment discontinuation in 9%. In 18% of the women with grade 3–4 toxicity, toxicity did not have any treatment consequences. Dose reduction due to any reason (grade 3–4 toxicity or other reasons) was seen in 72 (50%) patients, of which 76% were performed within the first three months. Treatment discontinuation due to toxicity was seen in 11 (8%) patients: discontinuation due to personal preference or disease-related symptoms in 7 (5%) patients. Unplanned hospitalization during treatment occurred in 19 (13%) patients, of which 4 (3%) were related to toxicity, 6 (4%) to disease progression and 9 (6%) due to other reasons. Of the 10 women who received a starting dose of 100 mg or 75 mg, 6 received another dose reduction, whereas dose was escalated to 125 mg in two participants.

### Associations between baseline characteristics and grade 3–4 toxicity

**Fig. 2:**
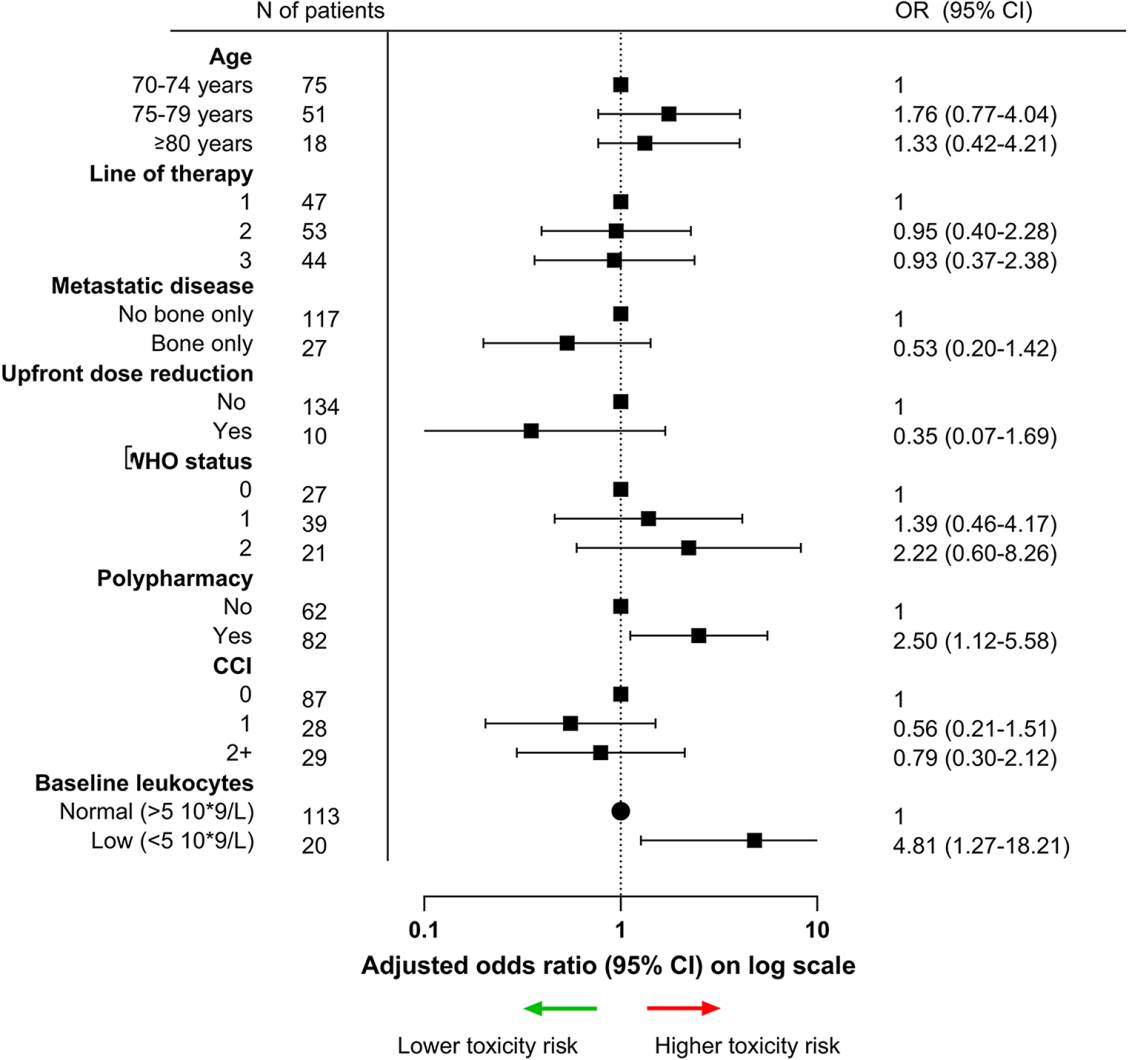
Forest plot of the multivariable logistic regression model to assess the association between baseline parameters and grade 3–4 palbociclib-related toxicity. Odds ratios (OR) and 95% confidence intervals (CI) are depicted. X-axis is displayed as log scale. Abbreviations: CCI; Charlson Comorbidity Index, CI: confidence interval, OR: odds ratio

In univariable logistic regression, pretreatment low leukocytes were associated with the development of grade 3–4 toxicity (OR 6.19; 95% CI 1.72–22.31, *p* = 0.005) (Table S2, Online Resource). After adjusting for bone only disease, line of therapy and WHO status in a multivariable regression model, low leukocytes (OR 4.81; 95% CI 1.27–18.21, *p* = 0.021) and polypharmacy (OR 2.50; 95% CI 1.12–5.58, *p* = 0.026) were associated with grade 3–4 toxicity, whereas a CCI of 1 (OR 0.56; 95% CI 0.21–1.51, *p* = 0.249), a CCI of ≥ 2 (OR 0.79; 95% CI 0.30–2.12, *p* = 0.643) and upfront dose reduction of palbociclib (OR 0.35, 95% CI 0.07–1.69, *p* = 0.191) were not associated with toxicity (Fig. 2*).*

### Effectiveness

**Fig. 3:**
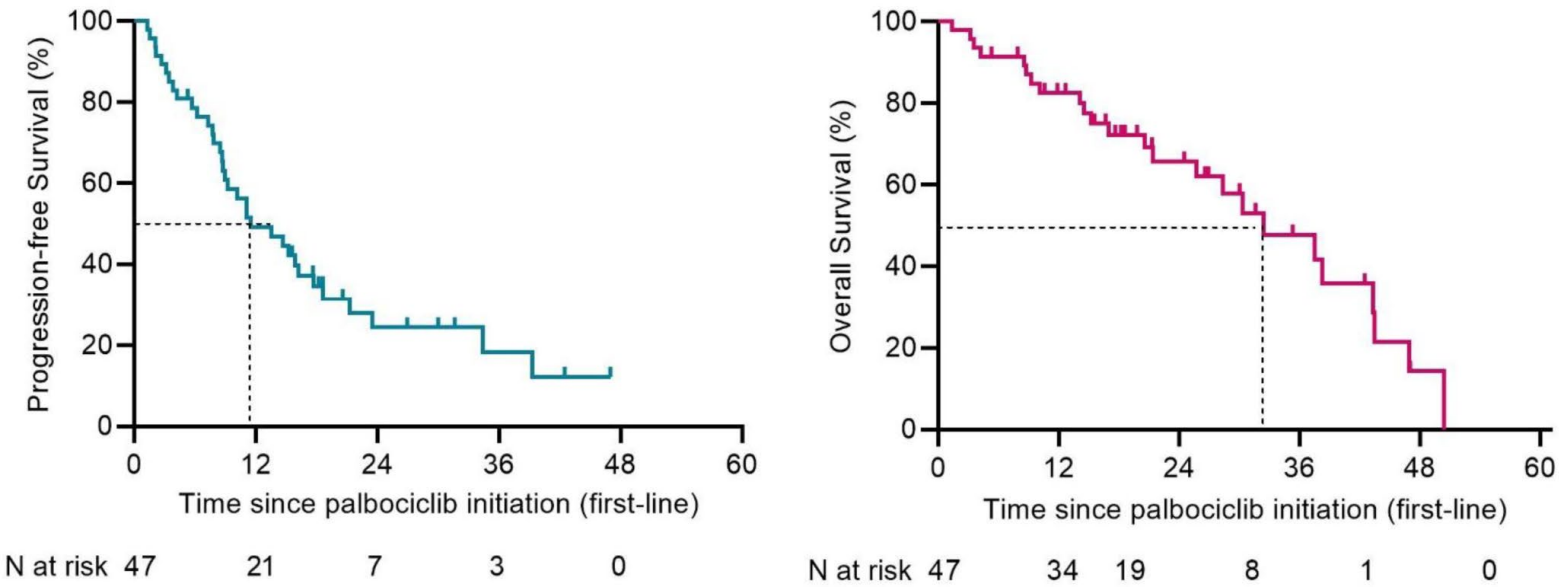
Kaplan-Meier survival plots of progression-free survival (PFS) and overall survival (OS) of those treated in the first-line setting. Median PFS was 11.5 months (95% CI 5.8-17.2) and median OS was 32.4 months (95% CI 21.8-43.1)

**Fig. 4:**
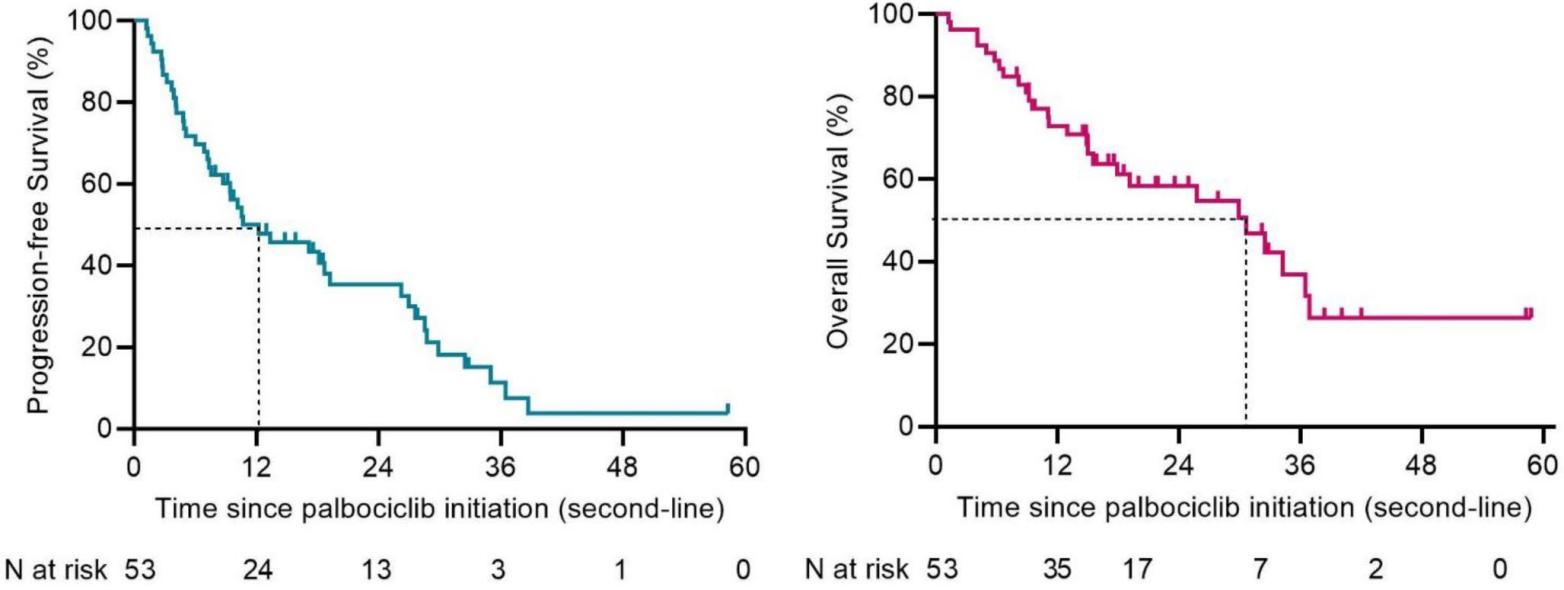
Kaplan-Meier survival plots of progression- free survival (PFS) and overall survival (OS) of those treated in the second-line setting. Median PFS was 12.2 (95% CI 4.1–20.4) and median OS 30.7 months (95% CI 17.1–44.3)

Baseline characteristics of women treated in the first- or second-line were comparable (Table S4). In women treated in the first-line systemic therapy, median PFS was 11.5 months (95% CI 5.8–17.2) and median OS 32.4 months (95% CI 21.8–43.1) (Fig. 3). For women treated in the second-line, median PFS was 12.2 (95% CI 4.1–20.4) and median OS 30.7 months (95% CI 17.1–44.3) (Fig. 4). In the third line or beyond, median PFS was 15.3 months (95% CI 8.3–22.4) and median OS 38.2 months (95% CI 22.8–53.7) (Figure S1, Online Resource).

### Sensitivity analysis

To investigate whether survival of women receiving upfront dose reduction or a dose reduction within the first three months was worse compared to those treated with the standard dose, we stratified survival analyses by dose reduction versus no dose reduction. Women in the dose-reduced group more often had recurrent or progressive disease and more often received prior chemotherapy, had a worse WHO status and more polypharmacy (Table S4, Online Resource). The median PFS of women who received a dose reduction was 15.9 months (95% CI 9.7–22.2), whereas women who did not receive a dose reduction had a median PFS of 11.1 months (95% CI 7.3–14.9) (log rank *p* = 0.71) (Figure S2, Online Resource). Median OS of those receiving dose-reduced treatment was 25.7 months (95% CI 19.2–33.2) and women receiving standard dose treatment had a median OS of 34.3 months (95% CI 29.4–39.1) (log rank *p* = 0.33) (Figure S2, Online Resource).

## Discussion

This real-world study showed that, despite the high occurrence of grade 3–4 neutropenia and dose reductions, only 13% of the older women treated with palbociclib developed severe nonhematological toxicity, 8% discontinued due to toxicity and 3% were hospitalized due to toxicity. Polypharmacy and low baseline leukocytes were associated with grade 3–4 toxicity, while comorbidities were not. Median PFS was 12 months and median OS 32 months in first-line, whereas median PFS was 12 months and median OS 31 months in second-line.

Interestingly, rates of grade 3–4 toxicities and neutropenia were slightly lower than reported in previous trials [17, 18] (Table 2). Reason for this discrepancy may be the relative higher percentage of our participants receiving an upfront dose reduction. Rates of nonhematological toxicity, dose reduction and dose delay were similar to previous studies [18–22]. Most toxicities could be adequately managed with dose adjustments and the low occurrence of hospitalization and toxicity-related discontinuation further reflects good tolerability in the older population. Previous studies with older women receiving CDK4/6 inhibitors showed that quality of life was maintained during treatment [18, 23], which is an important goal in treating metastatic breast cancer. Another argument in favor of CDK4/6 inhibitors is that it delays the use of chemotherapy [24], generally leading to more toxicity and decreased quality of life, especially in older patients [25]. Due to its favorable toxicity profile, palbociclib represent a valuable option in treating (frail) older women with metastatic breast cancer.

In our study, the effectiveness in first-line was substantially lower compared to the PALOMA-2 trial (median PFS of 25 months, median OS of 54 months) [9, 18, 19], which aligns with findings from other real-world studies [20, 26–28] (Table 2). This survival gap may partly be attributed to the strict protocol-specified definition of eligible patients for the PALOMA-2 trial, excluding individuals with poor performance status or extensive visceral disease. Furthermore, despite frail older adults making up a substantial proportion of the population receiving anticancer treatment [29, 30], their.

**Table 2:** Overview of previous studies investigating CDK4/6 inhibitors combined with endocrine therapy specifically in older women

	Study type	N of older patients	Grade 3+	Neutropenia gr 3+	Redu-ction	Delay	Disconti-nuation	Median PFS	Median OS
*Clinical trials*									
Rugo [18]	Pooled analysis trial	65-74y: 221 ≥ 75y: 83	65-74y: 78% 75+: 83%	65-74y: 63% 75+: 74%			6%	1 L: 28 months, 2 L:14–16 months	
Howie [17]	Pooled analysis trial	65-74y: 162 ≥ 75y: 56	88%				23%		
Malorni [31]	Phase II trial	115 (median age 67y)		72%	23%	35%	9%	2–4 L: 11 months	
*Real world data*									
Clifton [19]	Monocenter cohort	92			48%	57%		AL: 19 months	
Olazagasti [22]	Monocentercohort	73				38%	N/A	N/A	N/A
Gouton [20]	Monocenter cohort	52	67%	64%	40%		10%	AL: 9 months	NR
El Badri [21]	Multicenter cohort	≥ 75y: 276		46%	51%	59%	13%	1 L, 2 year: 65%	1 L, 2 year: 74%
Ismail [32]	DICA database	189 (median 64y)			39%			TTNT: 17 months	21 months
Patt [27]	Flatiron database	813 (median 65y)			35%		11%	1 L: 20 months	1 L: NR
DeMichele [33]	Flatiron database	772 (mean age 67y)						20 months	NR
Herrscher [34]	Monocenter cohort	77 (median 66y)		63%	31%		13%	2 L: 11 months 3–4 L: 9 months	NR
Rugo [35]	Flatiron database	≥ 65y: 450						1 L: 22 months	1 L: NR
Caillet [36]	Cohort	807	43%	32%	23%			N/A	N/A
Fountzilas [28]	Multicenter cohort	≥ 75y: 43	19%	19%	21%	17%		1 L: 11 months, 2 L: 8 months	1 L: 24 months, 2 L: NR
Our study	Multicenter cohort	144	54%	37%	50%	50%	8%	1 L: 12 months 2 L: 12 months	1 L: 32 months 2 L: 31 months

*Abbreviations* 1 L; first line, 2 L: second line, AL: any line of therapy, N/A: not applicable, NR: not reached, TTNT: time-to-next treatment

recruitment in trials remains challenging, even in studies with broad inclusion criteria [3, 37]. Irrespective of their eligibility status, patients with cancer who have comorbidities and those with an older age (frailty-related factors) are less frequently offered trial participation [38–40]. As a result, the selected trial population may have been diverse from the heterogenous and frail patient population treated in routine practice. This so-called efficacy-effectiveness gap is a commonly observed problem in oncology trials [41]. Lower treatment compliance, reduced tolerability and increased comorbidities of those treated in daily practice may diminish the magnitude of efficacy found in clinical trials. The fact that median OS in both arms of the PALOMA-2 trial was over 50 months [42], whereas real-world data in Dutch women with ER+/HER2- metastatic breast cancer treated with systemic therapy found a median OS of 33 months [43], suggests that the PALOMA-2 population had better patient- and tumor characteristics than those seen in daily practice.

Another reason for the different survival between trials and observational data might be that, in daily practice, women with the most aggressive tumor types and in highest need of rapid response were the first to be treated with this novel therapy of palbociclib in first-line. The Dutch Society of Medical Oncology has recommended second-line use of CDK4/6 inhibitors in patients with low-aggressive breast cancer, while awaiting the results of the Dutch SONIA trial on the preferred position of CDK4/6 inhibitor use [24, 44]. In this phase-3 randomized trial, the investigators evaluated the efficacy and safety of CDK4/6 inhibitors added to either first- or second-line endocrine therapy in patients with HR+/HER2 metastatic breast cancer [45]. Results from the SONIA study showed that first-line addition of CDK4/6 inhibitors did not provide a PFS benefit (time between randomization to second objective disease progression when CDK4/6 inhibitors were added in first-line was 31 months and added in second-line 28 months). However, first-line addition of CDK4/6 inhibitors did increase toxicity, suggesting that second-line use may indeed be the preferred option for most patients [46]: a potentially beneficial outcome for older patients.

Thirdly, during the COVID-19 pandemic, clinicians may have chosen only to add palbociclib in first-line in patients with the highest treatment urgency to avoid unnecessary hospital visits, and women with endocrine-sensitive tumor types and long-term response on first-line endocrine monotherapy may have received palbociclib in the second-line. More patients in the first-line setting received palliative radiotherapy and had ≥ 4 metastatic sites, which may indeed indicate that these patients more often had symptomatic or aggressive tumors.

Although women receiving dose reductions had a worse performance status and more polypharmacy, PFS was similar to those receiving the standard dose, which is in line with other studies in older women treated with palbociclib [32, 47]. Overall survival was slightly lower in women receiving a dose reduction, but this difference is more likely a result of patient selection rather than reduced response to palbociclib. Although these results are likely to be confounded by selection bias, they seem to be reassuring to clinicians and patients that de-escalation of treatment can be safely prescribed. Even though most grade 3–4 toxicities were manageable, they might lead to more frequent hospital visits and blood tests. Individuals at increased risk of developing toxicity, such patients with low leukocytes or polypharmacy, may therefore benefit from upfront dose reduction.

Although frailty status was not assessed in the current study, pretreatment frailty screening could aid physicians in further individualizing treatment with palbociclib in older women, as frailty is associated with an increased risk of poor treatment outcomes, functional decline and mortality [25, 29]. An evidence-based approach to diagnose frailty is by performing a geriatric assessment (GA) [48]. Although the role of a GA in palbociclib use is yet to be fully defined [49], geriatric characterization of older patients with metastatic breast cancer could help identify unmet needs and improve patient management, decision making and help maintaining quality of life [50]. Two ongoing prospective studies are currently investigating the association between geriatric questionnaires and treatment outcomes in older women treated with palbociclib [36, 51], which will help define the usefulness of a GA in clinical practice.

To our knowledge, this is the first real-world multicenter cohort that included women aged ≥ 70 years treated with palbociclib in any treatment line in both academic and community hospitals. Since all consecutive patients were included, we gathered a cohort of older women representable for daily practice. The high prevalence of multimorbidity and polypharmacy among our study population reflects the generalizability to patients seen in daily practice. Due to the relatively long follow-up period, this study was among the first to calculate median OS in older women.

Study limitations include the lack of a control group with women treated only with endocrine therapy to compare effectiveness, limited data about geriatric characteristics and quality of life and a modest sample size, especially in the subgroups stratified by line of therapy. Data extraction is dependent on the registration by clinicians in electronic health records, which may lead to incomplete data on patient characteristics, such as WHO performance status, or toxicity. Last, treatment outcomes might be influenced by interhospital variations in the performance of dose reductions.

## Conclusion

Although grade 3–4 toxicity and dose reductions occurred frequently, most were expected and managed by dose reductions, showing that palbociclib is generally well tolerated and thus represents a valuable treatment option in the older population.

### Electronic supplementary material

Below is the link to the electronic supplementary material.

Supplementary Material 1

## Data Availability

The datasets generated during and/or analysed during the current study are not publicly available due to participant privacy but are available from the corresponding author on reasonable request.

## References

[1] DeSantis CE, Ma J, Gaudet MM, Newman LA, Miller KD, Goding Sauer A (2019). Breast cancer statistics, 2019. CA Cancer J Clin.

[2] Zulman DM, Sussman JB, Chen X, Cigolle CT, Blaum CS, Hayward RA (2011). Examining the evidence: a systematic review of the inclusion and analysis of older adults in randomized controlled trials. J Gen Intern Med.

[3] van de Water W, Kiderlen M, Bastiaannet E, Siesling S, Westendorp RG, van de Velde CJ (2014). External validity of a trial comprised of elderly patients with hormone receptor-positive breast cancer. J Natl Cancer Inst.

[4] Rothwell PM (2005). External validity of randomised controlled trials: to whom do the results of this trial apply?. Lancet.

[5] Biganzoli L, Battisti NML, Wildiers H, McCartney A, Colloca G, Kunkler IH (2021). Updated recommendations regarding the management of older patients with breast cancer: a joint paper from the European Society of breast Cancer specialists (EUSOMA) and the International Society of Geriatric Oncology (SIOG). Lancet Oncol.

[6] Li J, Huo X, Zhao F, Ren D, Ahmad R, Yuan X (2020). Association of cyclin-dependent kinases 4 and 6 inhibitors with survival in patients with hormone receptor-positive metastatic breast Cancer: a systematic review and Meta-analysis. JAMA Netw Open.

[7] Cristofanilli M, Turner NC, Bondarenko I, Ro J, Im SA, Masuda N (2016). Fulvestrant plus palbociclib versus fulvestrant plus placebo for treatment of hormone-receptor-positive, HER2-negative metastatic breast cancer that progressed on previous endocrine therapy (PALOMA-3): final analysis of the multicentre, double-blind, phase 3 randomised controlled trial. Lancet Oncol.

[8] Finn RS, Crown JP, Lang I, Boer K, Bondarenko IM, Kulyk SO (2015). The cyclin-dependent kinase 4/6 inhibitor palbociclib in combination with letrozole versus letrozole alone as first-line treatment of oestrogen receptor-positive, HER2-negative, advanced breast cancer (PALOMA-1/TRIO-18): a randomised phase 2 study. Lancet Oncol.

[9] Finn RS, Martin M, Rugo HS, Jones S, Im SA, Gelmon K (2016). Palbociclib and Letrozole in Advanced breast Cancer. N Engl J Med.

[10] Turner NC, Slamon DJ, Ro J, Bondarenko I, Im SA, Masuda N (2018). Overall survival with Palbociclib and fulvestrant in advanced breast Cancer. N Engl J Med.

[11] Gennari A, Andre F, Barrios CH, Cortes J, de Azambuja E, DeMichele A (2021). ESMO Clinical Practice Guideline for the diagnosis, staging and treatment of patients with metastatic breast cancer. Ann Oncol.

[12] Corrigan-Curay J, Sacks L, Woodcock J (2018). Real-world evidence and real-World Data for evaluating Drug Safety and Effectiveness. JAMA.

[13] Charlson ME, Pompei P, Ales KL, MacKenzie CR (1987). A new method of classifying prognostic comorbidity in longitudinal studies: development and validation. J Chronic Dis.

[14] Nilsson G, Hedberg P, Ohrvik J (2014). White blood cell count in elderly is clinically useful in predicting long-term survival. J Aging Res.

[15] Services USDoHaH (2017) Common Terminology Criteria for Adverse Events (CTCAE), Version 5.0

[16] Shuster JJ (1991). Median follow-up in clinical trials. J Clin Oncol.

[17] Howie LJ, Singh H, Bloomquist E, Wedam S, Amiri-Kordestani L, Tang S (2019). Outcomes of older women with hormone Receptor-Positive, human epidermal growth factor receptor-negative metastatic breast Cancer treated with a CDK4/6 inhibitor and an aromatase inhibitor: an FDA pooled analysis. J Clin Oncol.

[18] Rugo HS, Turner NC, Finn RS, Joy AA, Verma S, Harbeck N (2018). Palbociclib plus endocrine therapy in older women with HR+/HER2- advanced breast cancer: a pooled analysis of randomised PALOMA clinical studies. Eur J Cancer.

[19] Clifton K, Min Y, Kimmel J, Litton J, Tripathy D, Karuturi M (2019). Progression-free survival (PFS) and toxicities of palbociclib in a geriatric population. Breast Cancer Res Treat.

[20] Gouton E, Tassy L, Micallef J, Meskine A, Sabatier R, Cecile-Herry M et al (2022) The safety and efficacy of palbociclib in older patients with advanced breast cancer in a real-world setting. J Cancer Metas Treat. ;8

[21] El Badri S, Tahir B, Balachandran K, Bezecny P, Britton F, Davies M (2021). Palbociclib in combination with aromatase inhibitors in patients >/= 75 years with oestrogen receptor-positive, human epidermal growth factor receptor 2 negative advanced breast cancer: a real-world multicentre UK study. Breast.

[22] Olazagasti C, Lee CS, Liu A, Stefanov D, Cheng K (2023). A deep dive into CDK4/6 inhibitors: evaluating real world toxicities and treatment paradigms in the elderly population. J Oncol Pharm Pract.

[23] Di Lauro V, Barchiesi G, Martorana F, Zucchini G, Muratore M, Fontanella C (2022). Health-related quality of life in breast cancer patients treated with CDK4/6 inhibitors: a systematic review. ESMO Open.

[24] Meegdes M, Geurts SME, Erdkamp FLG, Dercksen MW, Vriens B, Aaldering KNA (2022). The implementation of CDK 4/6 inhibitors and its impact on treatment choices in HR+/HER2- advanced breast cancer patients: a study of the Dutch SONABRE Registry. Int J Cancer.

[25] Baltussen JC, de Glas NA, van Holstein Y, van der Elst M, Trompet S, Uit den Boogaard A (2023). Chemotherapy-related toxic effects and Quality of Life and Physical Functioning in older patients. JAMA Netw Open.

[26] Cardoso Borges F, Alves da Costa F, Ramos A, Ramos C, Bernardo C, Brito C (2022). Real-world effectiveness of palbociclib plus fulvestrant in advanced breast cancer: results from a population-based cohort study. Breast.

[27] Patt D, Liu X, Li B, McRoy L, Layman RM, Brufsky A (2022). Real-world treatment patterns and outcomes of Palbociclib plus an aromatase inhibitor for metastatic breast Cancer: Flatiron Database Analysis. Clin Breast Cancer.

[28] Fountzilas E, Koliou GA, Vozikis A, Rapti V, Nikolakopoulos A, Boutis A et al (2020) Real-world clinical outcome and toxicity data and economic aspects in patients with advanced breast cancer treated with cyclin-dependent kinase 4/6 (CDK4/6) inhibitors combined with endocrine therapy: the experience of the Hellenic Cooperative Oncology Group. ESMO Open. ;5 10.1136/esmoopen-2020-000774 PMC7437702 32817060

[29] Handforth C, Clegg A, Young C, Simpkins S, Seymour MT, Selby PJ (2015). The prevalence and outcomes of frailty in older cancer patients: a systematic review. Ann Oncol.

[30] Wang S, Yang T, Qiang W, Shen A, Zhao Z, Yang H (2022). The prevalence of frailty among breast cancer patients: a systematic review and meta-analysis. Support Care Cancer.

[31] Malorni L, Curigliano G, Minisini AM, Cinieri S, Tondini CA, D’Hollander K (2018). Palbociclib as single agent or in combination with the endocrine therapy received before disease progression for estrogen receptor-positive, HER2-negative metastatic breast cancer: TREnd trial. Ann Oncol.

[32] Ismail RK, van Breeschoten J, Wouters M, van Dartel M, van der Flier S, Reyners AKL (2021). Palbociclib dose reductions and the effect on clinical outcomes in patients with advanced breast cancer. Breast.

[33] DeMichele A, Cristofanilli M, Brufsky A, Liu X, Mardekian J, McRoy L (2021). Comparative effectiveness of first-line palbociclib plus letrozole versus letrozole alone for HR+/HER2- metastatic breast cancer in US real-world clinical practice. Breast Cancer Res.

[34] Herrscher H, Velten M, Leblanc J, Kalish-Weindling M, Fischbach C, Exinger D (2020). Fulvestrant and palbociclib combination in heavily pretreated hormone receptor-positive, HER2-negative metastatic breast cancer patients. Breast Cancer Res Treat.

[35] Rugo HS, Liu X, Li B, McRoy L, Layman RM, Brufsky A (2023). Real-world comparative effectiveness of palbociclib plus letrozole versus letrozole in older patients with metastatic breast cancer. Breast.

[36] Caillet P, Pulido M, Brain E, Falandry C, Desmoulins I, Ghebriou D (2021). PALOMAGE, a French real-world cohort of elderly women beyond age 70 with advanced breast cancer receiving palbociclib: baseline characteristics and safety evaluation. J Clin Oncol.

[37] Habib MH, Alibhai SMH, Puts M (2024). How representative are participants in geriatric oncology clinical trials? The case of the 5 C RCT in geriatric oncology: a cross-sectional comparison to a geriatric oncology clinic. J Geriatr Oncol.

[38] Unger JM, Hershman DL, Fleury ME, Vaidya R (2019). Association of Patient Comorbid conditions with Cancer Clinical Trial Participation. JAMA Oncol.

[39] Brooks SE, Carter RL, Plaxe SC, Basen-Engquist KM, Rodriguez M, Kauderer J (2015). Patient and physician factors associated with participation in cervical and uterine cancer trials: an NRG/GOG247 study. Gynecol Oncol.

[40] Kemeny MM, Peterson BL, Kornblith AB, Muss HB, Wheeler J, Levine E (2003). Barriers to clinical trial participation by older women with breast cancer. J Clin Oncol.

[41] Di Maio M, Perrone F, Conte P (2020). Real-world evidence in Oncology: opportunities and limitations. Oncologist.

[42] Finn RS, Rugo HS, Dieras VC, Harbeck N, Im S-A, Gelmon KA (2022). Overall survival (OS) with first-line palbociclib plus letrozole (PAL + LET) versus placebo plus letrozole (PBO + LET) in women with estrogen receptor–positive/human epidermal growth factor receptor 2–negative advanced breast cancer (ER+/HER2 – ABC): analyses from PALOMA-2. J Clin Oncol.

[43] Meegdes M, Geurts SME, Erdkamp FLG, Dercksen MW, Vriens B, Aaldering KNA (2023). Real-world time trends in overall survival, treatments and patient characteristics in HR+/HER2- metastatic breast cancer: an observational study of the SONABRE Registry. Lancet Reg Health Eur.

[44] Honkoop AHB H.J. Plaatsbepaling NABON en NVMO: toepassing van palbociclib bij mammacarcinoom. Medische Oncol2017

[45] van Ommen-Nijhof A, Konings IR, van Zeijl CJJ, Uyl-de Groot CA, van der Noort V, Jager A (2018). Selecting the optimal position of CDK4/6 inhibitors in hormone receptor-positive advanced breast cancer - the SONIA study: study protocol for a randomized controlled trial. BMC Cancer.

[46] Nijhof AVO, Wortelboer N, Noort Vvd, Swinkels ACP, Blommestein HM, Beeker A (2023). Primary outcome analysis of the phase 3 SONIA trial (BOOG 2017-03) on selecting the optimal position of cyclin-dependent kinases 4 and 6 (CDK4/6) inhibitors for patients with hormone receptor-positive (HR+), HER2-negative (HER2-) advanced breast cancer (ABC). J Clin Oncol.

[47] Zheng J, Yu Y, Durairaj C, Dieras V, Finn RS, Wang DD (2021). Impact of dose reduction on efficacy: implications of exposure-response analysis of Palbociclib. Target Oncol.

[48] Jones D, Song X, Mitnitski A, Rockwood K (2005). Evaluation of a frailty index based on a comprehensive geriatric assessment in a population based study of elderly canadians. Aging Clin Exp Res.

[49] Battisti NML, De Glas N, Sedrak MS, Loh KP, Liposits G, Soto-Perez-de-Celis E (2018). Use of cyclin-dependent kinase 4/6 (CDK4/6) inhibitors in older patients with ER-positive HER2-negative breast cancer: Young International Society of Geriatric Oncology review paper. Ther Adv Med Oncol.

[50] Wildiers H, Heeren P, Puts M, Topinkova E, Janssen-Heijnen ML, Extermann M (2014). International Society of Geriatric Oncology consensus on geriatric assessment in older patients with cancer. J Clin Oncol.

[51] Tripathy D, Blum JL, Rocque GB, Bardia A, Karuturi MS, Cappelleri JC (2020). POLARIS: a prospective, multicenter, noninterventional study assessing palbociclib in hormone receptor-positive advanced breast cancer. Future Oncol.

