# Supplementary material for: Tolerability and effectiveness of palbociclib in older women with metastatic breast cancer
Source: Breast Cancer Res Treat. 2024 Apr 16;206(2):337–46. doi: 10.1007/s10549-024-07312-y (PMC11182846; doi:10.1007/s10549-024-07312-y)
Supplement: Supplementary file 1 — Supplementary Material 1 [file 10549_2024_7312_MOESM1_ESM.docx]

**Appendix: Tolerability and effectiveness of palbociclib in older women with metastatic breast cancer**

*Breast Cancer Research and Treatment*

**Authors**

Joosje C. Baltussen^1^, Simon P. Mooijaart^2,3^, Annelie J.E. Vulink^4^, Danny Houtsma^5^, Wendy M. Van der Deure^6^, Elsbeth M. Westerman^7^, Hendrika M. Oosterkamp^8^, Leontine E.A.M.M. Spierings^9^, Frederiek van den Bos ^2,3^, Nienke A. de Glas^1^, Johanneke E.A. Portielje^1^

**Affiliations**^1^ Department of Medical Oncology, Leiden University Medical Center, Leiden, the Netherlands

^2^Department of Gerontology and Geriatrics, Leiden University Medical Center, Leiden, the Netherlands

^3^LUMC Center for Medicine for Older People, LUMC, Leiden, The Netherlands

^4^Department of Medical Oncology, Reinier de Graaf Hospital, Delft, the Netherlands

^5^Department of Internal Medicine, Haga Hospital, The Hague, the Netherlands

^6^Department of Internal Medicine, Groene Hart Hospital, Gouda, the Netherlands

^7^Department of Clinical Pharmacy, Haaglanden Medical Center, The Haque, The Netherlands

^8^Department of Medical Oncology, Haaglanden Medical Center, The Haque, The Netherlands

^9^Department of Internal Medicine, Alrijne Hospital, Leiderdorp, The Netherlands

**Email address Corresponding Author:** J.C.Baltussen@lumc.nl

| Table S1: Treatment-related toxicities |  |
| --- | --- |
|  | Grade 3-4 toxicity (%) |
| Hematological | **63 (43.8)** |
| Neutropenia | 55 (38.2) |
| Febrile neutropenia | 0 (0) |
| Leukopenia | 10 (6.9) |
| Anemia | 4 (2.8) |
| Thrombopenia | 2 (1.4) |
| Non-hematological | **18 (12.5)** |
| Fatigue | 9 (6.2) |
| Nausea/vomiting | 4 (2.8) |
| Infection without neutropenia | 2 (1.4) |
| Mucositis | 3 (2.1) |

| Table S2: Associations between baseline characteristics and grade 3-4 toxicity | | | | | | |
| --- | --- | --- | --- | --- | --- | --- |
| Variable | Category | N of patients | Univariable (OR, 95% CI) | p-value | Multivariable (OR, 95% CI) | p-value |
| Age (years) | 70-74 | 75 | Ref |  | Ref |  |
|  | 75-79 | 51 | 1.99 (0.96-4.13) | .066 | 1.76 (0.77-4.04) | .179 |
|  | ≥ 80 | 18 | 1.08 (0.39-3.02) | .525 | 1.33 (0.42-4.21) | .633 |
| Metastatic | No bone only | 117 | Ref |  | Ref |  |
| disease | Bone only | 27 | 0.74 (0.32-1.72) | .487 | 0.53 (0.20-1.42) | .210 |
| Line of | 1 | 47 | Ref |  | Ref |  |
| therapy | 2 | 53 | 1.06 (0.48-2.34) | .879 | 0.95 (0.40-2.28) | .908 |
|  | 3 | 44 | 1.06 (0.46-2.41) | .897 | 0.93 (0.37-2.38) | .881 |
| Upfront | No | 134 | Ref |  | Ref |  |
| reduction | Yes | 10 | 0.34 (0.08-1.36) | .127 | 0.35 (0.07-1.69) | .191 |
| Baseline leukocytes | >5 10*9/L  ≤5 10*9/L  Unknown | 113  20  11 | Ref  6.19 (1.72-22.31)  1.92 (0.53-6.90) | .005  .322 | Ref  4.81 (1.27-18.21)  2.45 (0.57-10.54) | .021  .228 |
| WHO status | 0 | 27 | Ref |  | Ref |  |
|  | 1 | 41 | 1.02 (0.38-2.73) | .964 | 1.39 (0.46-4.17) | .560 |
|  | 2 | 23 | 1.75 (0.55-5.58) | .344 | 2.22 (0.60-8.26) | .235 |
|  | Unknown | 59 | 1.48 (0.59-3.72) | .403 | 1.57 (0.54-4.56) | .407 |
| Polypharmacy | No | 62 | Ref |  | Ref |  |
|  | Yes | 82 | 1.89 (0.93-3.70) | .060 | 2.50 (1.12-5.58) | .026 |
| CCI | 0 | 87 | Ref |  | Ref |  |
|  | 1 | 28 | 0.85 (0.36-2.00) | .711 | 0.56 (0.21-1.51) | .249 |
|  | 2 or more | 29 | 1.21 (0.52-2.83) | .667 | 0.79 (0.30-2.12) | .643 |
| Living | With others | 66 | Ref |  |  |  |
| situation | Alone | 54 | 0.80 (0.39-1.65) | .550 |  |  |
|  | Institutionalized | 4 | 2.53 (0.25-25.49) | .432 |  |  |

Uni- and multivariate logistic regression for the association between baseline characteristics and CDK4/6 inhibitor-related grade 3-4 toxicity. Clinically relevant (age, metastatic disease, line of therapy, upfront dose reduction, WHO status, CCI) and significant (p<0.1) predictors were included in the multivariable analysis.
Abbreviations: CCI; Charlson Comorbidity Index, OR: odds ratio.

| Table S3: Baseline characteristics, stratified for first- or second-line therapy | | | |  |
| --- | --- | --- | --- | --- |
|  | Variable | First-line (N=47) | Second-line (N=53) | p-value* |
| Age | 70-74 | 29 (62) | 24 (45) | .240 |
|  | 75-79 | 14 (30) | 21 (40) |  |
|  | ≥ 80 | 4 (9) | 8 (15) |  |
| Presentation | Newly diagnosed disease | 16 (34) | 15 (29) | .536 |
|  | Recurrent or progressive disease | 31 (66) | 38 (71) |  |
| Metastases | Bone only | 6 (13) | 10 (19) | .406 |
|  | No bone only | 41 (87) | 43 (81) |  |
| Number of | 1 | 10 (21) | 14 (26) | .280 |
| Metastatic sites | 2 | 18 (38) | 22 (42) |  |
|  | 3 | 12 (26) | 15 (28) |  |
|  | 4 or more | 7 (15) | 2 (4) |  |
| Starting dose | Standard dose | 43 (92) | 50 (94) | .577 |
|  | Upfront dose reduction | 4 (9) | 3 (6) |  |
| Prior chemotherapy | Yes | 5 (11) | 2 (4) | .217 |
| Concurrent radiotherapy | Yes | 12 (26) | 7 (13) | .111 |
| WHO performance status | 0  1  ≥2  Not recorded | 8 (17)  14 (30)  9(19)  16 (34) | 12 (23)  14 (26)  8 (15)  19 (36) | .859 |
| Charlson | 0 | 27 (57) | 33 (62) | .252 |
| Comorbidity Index | 1 | 15 (32) | 9 (17) |  |
|  | 2 | 4 (9) | 8 (15) |  |
|  | 3 | 1 (2) | 3 (6) |  |
| N of medications | 0-4 | 19 (40) | 22 (42) | .750 |
|  | ≥5 | 28 (60) | 31 (58) |  |
| Living situation | With others | 26 (55) | 25 (47) | .325 |
|  | Alone | 15 (32) | 23 (43) |  |
|  | Institutionalized | 2 (4) | 0 |  |
|  | Unknown | 4 (9) | 5 (9) |  |
| Treatment duration (months) | Median (IQR) | 10 (5-18) | 9 (4-18) | .858 |

*p-value represents the Chi-square test for categorical variables or independent t-test for continuous variables.

| Table S4: Baseline characteristics, stratified for dose reduction before or during treatment | | | |  |
| --- | --- | --- | --- | --- |
|  | Variable | No reduction (N=85) | Reduction (N=59) | p-value* |
| Age | 70-74 | 48 (57) | 27 (46) | .421 |
|  | 75-79 | 28 (33) | 23 (39) |  |
|  | ≥ 80 | 9 (11) | 9 (15) |  |
| Presentation | Newly diagnosed disease | 28 (33) | 11 (19) | .058 |
|  | Recurrent or progressive disease | 57 (67) | 48 (81) |  |
| Metastases | Bone only | 14 (16) | 13 (22) | .400 |
|  | No bone only | 71 (84) | 46 (78) |  |
| Endocrine therapy | Aromatase inhibitor | 24 (28) | 17 (29) | .940 |
|  | Anti-estrogen | 61 (72) | 42 (72) |  |
| Line of endocrine therapy | 1 | 32 (38) | 15 (24) | .214 |
|  | 2 | 31 (37) | 22 (37) |  |
|  | ≥3 | 22 (26) | 23 (37) |  |
| Prior chemotherapy | Yes | 6 (7) | 10 (17) | .073 |
| Radiotherapy | Yes | 11 (13) | 11 (19) | .350 |
| WHO performance status | 0  1  ≥2  Not recorded | 19 (22)  22 (26)  7 (8)  37 (44) | 8 (14)  17 (29)  14 (24)  20 (34) | .044 |
| Charlson | 0 | 51 (60) | 38 (64) | .479 |
| Comorbidity | 1 | 20 (24) | 8 (14) |  |
| Index | 2 | 10 (11) | 9 (15) |  |
|  | 3 | 4 (5) | 4 (7) |  |
| N of medications | 0-4 | 42 (49) | 20 (34) | .064 |
|  | ≥5 | 43 (51) | 39 (66) |  |
| Living situation | With others | 42 (49) | 24 (41) | .685 |
|  | Alone | 31 (37) | 24 (41) |  |
|  | Institutionalized | 2 (2) | 1 (2) |  |
|  | Unknown | 10 (12) | 10 (17) |  |

*p-value represents the Chi-square test for categorical variables.


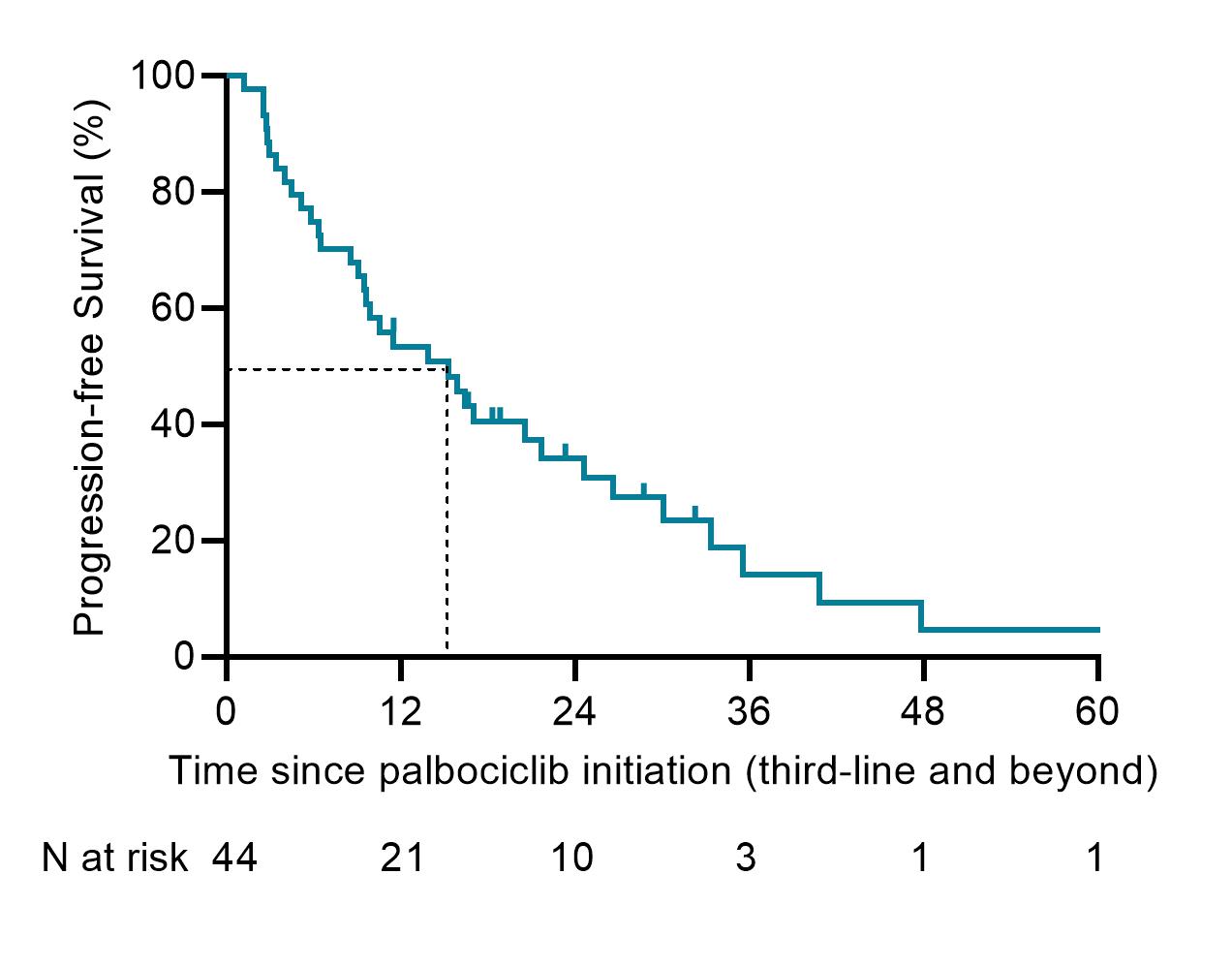


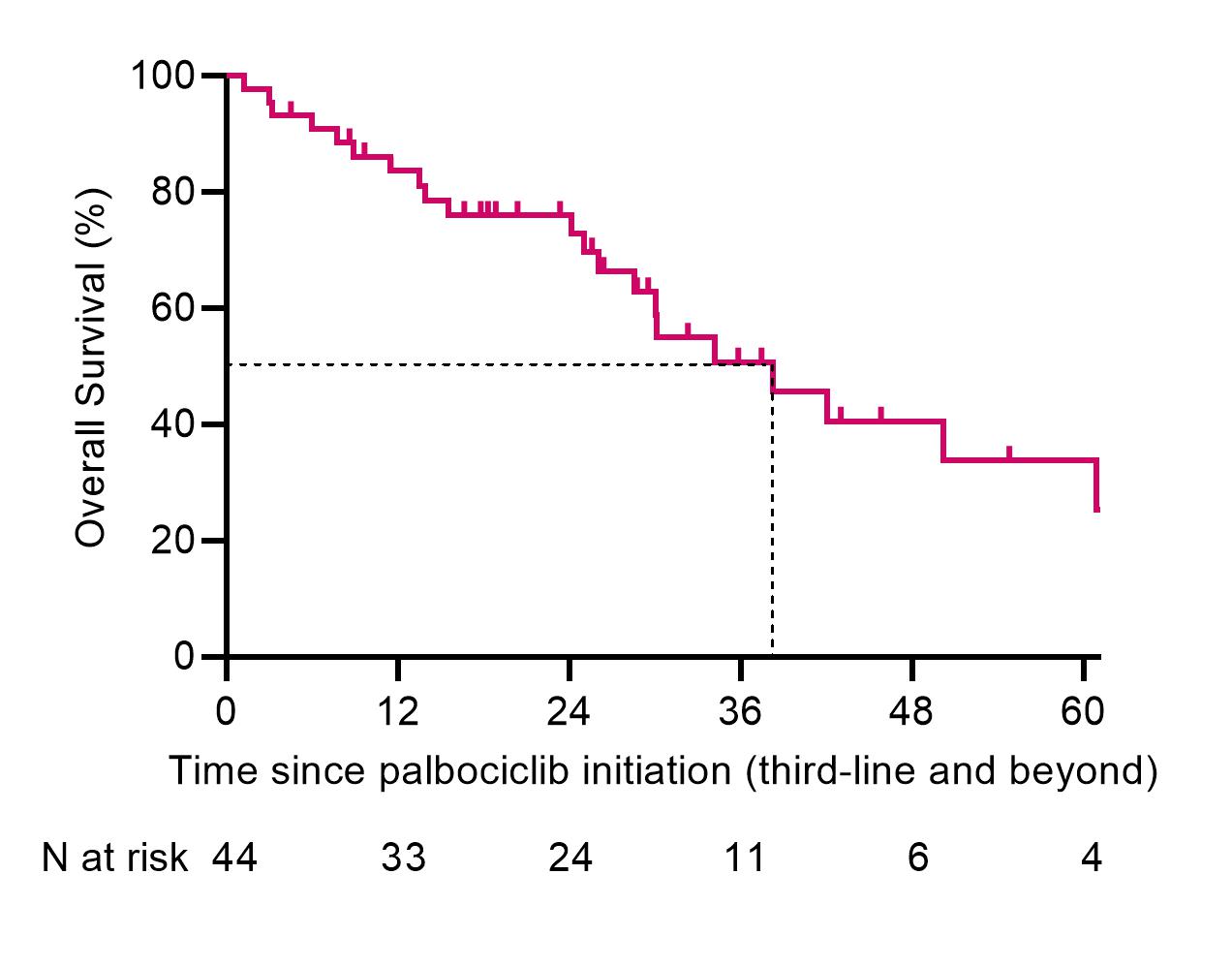


Figure S1: Kaplan-Meier survival plots showing the progression-free survival (PFS) and overall survival (OS) of patients treated in the third-line or beyond. Median PFS was 15.3 months (95% CI 8.3-22.4) and median OS 38.2 months (95% CI 22.8-53.7)


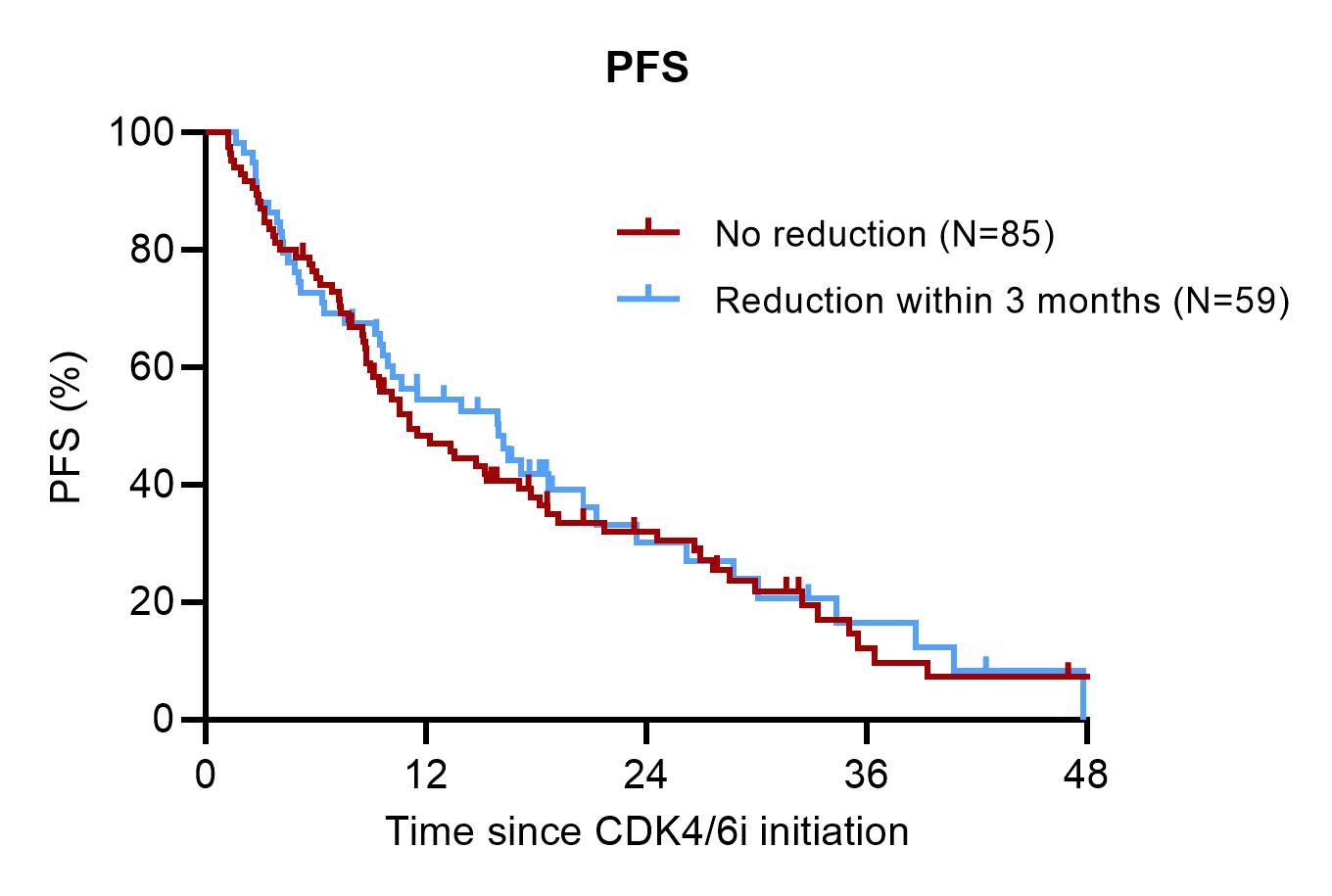


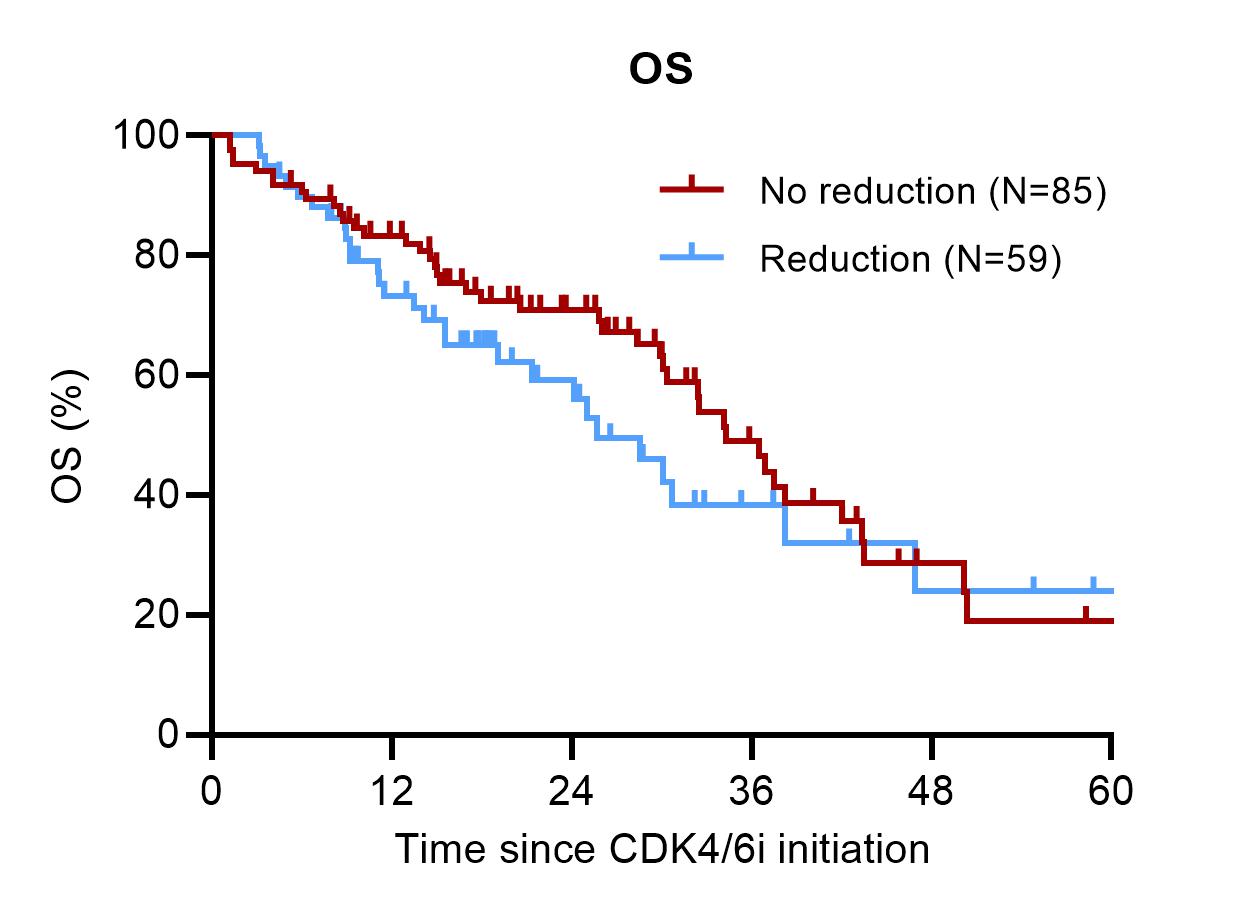


Figure S2: Kaplan-Meier survival plots showing the progression-free survival (PFS) and overall survival (OS) of patients, stratified by dose reduction before treatment or during the first three months of treatment. Log-rank test between two groups: PFS p=0.71, OS p=0.33.
